# Nursery origin of yellowfin tuna in the western Atlantic Ocean: significance of Caribbean Sea and trans-Atlantic migrants

**DOI:** 10.1038/s41598-023-43163-1

**Published:** 2023-09-28

**Authors:** Jay R. Rooker, Michelle Zapp Sluis, Larissa L. Kitchens, Michael A. Dance, Brett Falterman, Jessica M. Lee, Hui Liu, Nathaniel Miller, Hilario Murua, Alexandra M. Rooker, Eric Saillant, John Walter, R. J. David Wells

**Affiliations:** 1 Department of Marine Biology, Texas A&M University at Galveston, Galveston, TX 77553 USA; 2 Department of Ecology and Conservation Biology, Texas A&M University, College Station, TX 77843 USA; 3 Department of Oceanography and Coastal Sciences, Louisiana State University, Baton Rouge, LA 70803 USA; 4 Fisheries Research Support, Mandeville, LA 70448 USA; 5 Jackson School of Geosciences, The University of Texas at Austin, Austin, TX 78712 USA; 6 International Seafood Sustainability Foundation, Pittsburgh, PA 15201-1820 USA; 7 AZTI Marine Research, Basque Research and Technology Alliance (BRTA), Pasaia, Spain; 8 School of Ocean Science and Engineering, University of Southern Mississippi, Ocean Springs, MS 39564 USA; 9 Southeast Fisheries Science Center, NOAA Fisheries, Miami, FL 33149 USA

**Keywords:** Animal migration, Fisheries, Ecology, Ecology, Ocean sciences

## Abstract

Natural geochemical markers in the otolith of yellowfin tuna (*Thunnus albacares*) were used to establish nursery-specific signatures for investigating the origin of fish captured in the western Atlantic Ocean (WAO). Two classes of chemical markers (trace elements, stable isotopes) were used to first establish nursery-specific signatures of age-0 yellowfin tuna from four primary production zones in the Atlantic Ocean: Gulf of Mexico, Caribbean Sea, Cape Verde, and Gulf of Guinea. Next, mixture and individual assignment methods were applied to predict the origin of sub-adult and adult yellowfin tuna from two regions in the WAO (Gulf of Mexico, Mid Atlantic Bight) by relating otolith core signatures (corresponding to age-0 period) to baseline signatures of age-0 fish from each nursery. Significant numbers of migrants from Caribbean Sea and eastern Atlantic Ocean (EAO) production zones (Gulf of Guinea, Cape Verde) were detected in the WAO, suggesting that fisheries in this region were subsidized by outside spawning/nursery areas. Contributions from local production (Gulf of Mexico) were also evident in samples from both WAO fisheries, but highly variable from year to year. High levels of mixing by yellowfin tuna from the different production zones and pronounced interannual trends in nursery-specific contribution rates in the WAO emphasize the complex and dynamic nature of this species’ stock structure and population connectivity. Given that geographic shifts in distribution across national or political boundaries leads to governance and management challenges, this study highlights the need for temporally resolved estimates of nursery origin to refine assessment models and promote the sustainable harvest of this species.

## Introduction

Tropical and temperate tunas are found throughout the world’s oceans and represent essential components of pelagic ecosystems. As higher order consumers, tunas influence the population dynamics and community structure of prey assemblages, primarily through top-down control^1, 2^. True tunas (Genus *Thunnus*) including albacore, bluefin, yellowfin, and bigeye are highly migratory by nature and commonly traverse international borders and management boundaries^3^. Still, restricted movements and high site fidelity to specific basins or regions have also been reported for subpopulations or stocks^4, 5^. Conservation efforts are fundamentally influenced by migratory patterns of tunas because directed fishing, bycatch, and environmental stressors vary both spatially and temporally across a species’ geographic range^6^. As a result, an improved understanding of the migration ecology of tunas is increasingly implicated as a data gap that compromises their sustainable management^7–10^.

In the Atlantic Ocean, yellowfin tuna (*Thunnus albacares*) support important commercial and recreational fisheries^11^. Recent assessments by the International Commission for the Conservation of Atlantic Tunas (ICCAT) indicate that the Atlantic population is not overfished; however, a continuous decline in yellowfin tuna biomass relative to the biomass to produce maximum sustainable yield (B_MSY_) over time suggests the population may not be sustainable at current rates of exploitation^12, 13^. Management of yellowfin tuna is further complicated by the fact that data on their migrations to and from spawning and nursery areas are extremely limited. Several spawning areas exist for yellowfin tuna in the Atlantic Ocean, but the largest production zone is centered in equatorial waters off Africa in the Gulf of Guinea^13, 14^. An additional spawning and/or nursery area in the east occurs farther to the north near Cape Verde^13, 15^, while other notable spawning and nursery areas are present farther west in the Caribbean Sea and Gulf of Mexico^16–20^. Findings from both tagging^3^ and genetic^21^ studies suggest some degree of stock mixing exists among migrants from the different spawning areas in the Atlantic Ocean; however, the magnitude of trans-Atlantic migrations and the degree of mixing by migrants from different production zones is currently unknown.

Here, we applied otolith geochemistry to estimate the origin and mixing rates of yellowfin tuna in regional fisheries in the western Atlantic Ocean (WAO). A preliminary study assessed the potential of using chemical markers in otoliths of age-0 or young-of-the-year yellowfin tuna to distinguish individuals from different production zones^15^. The current research builds on this earlier study by establishing a comprehensive baseline sample of “nursery-specific” geochemical signatures of age-0 yellowfin tuna from the four primary production zones in the Atlantic Ocean (Gulf of Mexico, Caribbean Sea, Cape Verde, Gulf of Guinea) over a five-year period (2013–2017). Next, the nursery origin of sub-adult and adult yellowfin tuna from fisheries in the Gulf of Mexico and Mid Atlantic Bight was determined by comparing geochemical signatures in their otolith cores (corresponds to age-0 period) to age-0 baselines using two mixed-stock approaches (HISEA and random forest). The aim of this study was to determine whether WAO fisheries are supported by local production or subsidized by contributions from outside production zones in the Caribbean Sea and eastern Atlantic Ocean (EAO).

## Methods

**Figure 1:**
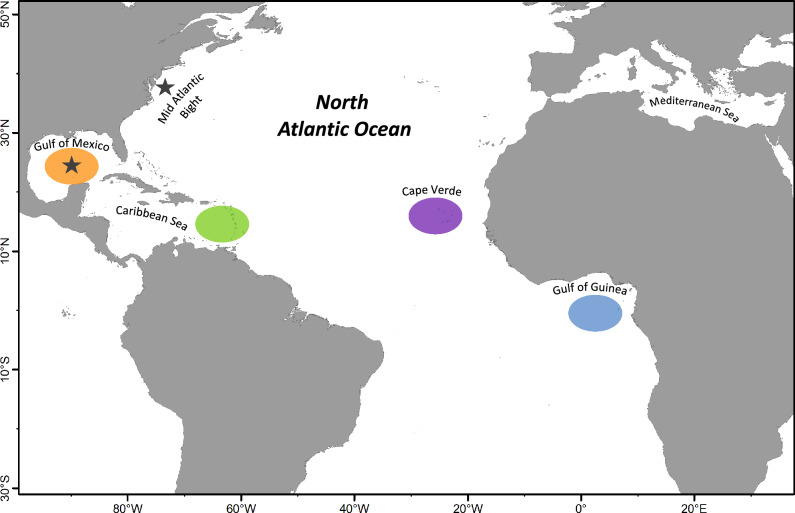
Map showing the four regional nurseries sampled for age-0 yellowfin tuna (*Thunnus albacares*) in the Atlantic Ocean from 2013 to 2017: Gulf of Mexico (orange), Caribbean Sea (green), Cape Verde (purple), and Gulf of Guinea (blue). Sub-adult and adult yellowfin tuna of unknown origin were also sampled from two locations (star symbol) in the western Atlantic Ocean (WAO): Gulf of Mexico and Mid Atlantic Bight. Map generated with QGIS (V. 3.32, www.qgis.org).

Baseline samples used to establish region-specific geochemical signatures were comprised of age-0 individuals collected from the four putative spawning and/or nursery areas in the Atlantic Ocean: (1) Gulf of Mexico, (2) Windward Island chain in the Caribbean Sea (hereafter Caribbean Sea), (3) Cape Verde, and (4) Gulf of Guinea (Fig. 1). Age-0 yellowfin tuna were captured yearly from 2013 to 2017 to obtain samples from each of the four geographic regions every year. This was required to develop baselines for each age-0 cohort, which allowed for age-class matching the birth year of adults to the correct age-0 baseline sample. Due to sampling limitations, samples from 2015 and 2016 were pooled for each region to develop a baseline for this period. The smallest age-0 fish available (< 45 cm FL) were targeted during all collections from each geographic region to minimize the potential for movements between geographic zones prior to collection. Yellowfin tuna in age-0 baseline samples were approximately 3–8 months old (range/mean = 24–55/39 cm FL), and all fish were less than 1 year old (ca. < 55 cm FL).

Sub-adult and adult yellowfin tuna (ca. age-1 to 3 +, range/mean = 65–128/89 cm FL; hereafter adult) were collected in 2014, 2015, 2016, and 2017 from two geographic regions of the WAO: (1) Gulf of Mexico and (2) Mid Atlantic Bight. Adults were captured with hook-and-line gear by both recreational and commercial anglers. Since the intention was to match birth year of each adult (unknown origin) with the correct baseline year, only yellowfin tuna determined to be age-4 or less were used for mixed-stock analysis. Age determination was based on direct aging or estimated from length using an age-length key^22^. Apart from archived samples from other institutions, all collections of age-0 and adult tuna were in accordance with institutional (Texas A&M University) and ARRIVE guidelines and, as required, covered under NOAA Fisheries exempted fishing permits (EFPs) to JRR (TUNA-EFP-13-03, HMS-EFP-14-02, HMS-EFP-15-02). Limited sampling (wild captures) of juvenile yellowfin tuna in the Gulf of Mexico was conducted under approved animal study protocol by the Institutional Animal Care and Use Committee at Texas A&M University (TAMU IACUC 2014-0026).

Sagittal otoliths of age-0 and adult yellowfin tuna were extracted, cleansed of adhering tissue, rinsed with deionized water (DIH_2_O), and stored dry in plastic vials. One otolith from each specimen was embedded in EpoFix resin (Struers A/S) and sectioned using a low-speed ISOMET saw (Beuhler) to obtain a 1.0 mm section of the core of the otolith, following established protocols^9^. Thin sections were mounted onto glass slides using Crystalbond thermoplastic glue (SPI Supplies/Structure Probe Inc.) and polished using 0.3 mm MicroPolish Alumina Powder and 600–1200 grit silicone-carbide paper (Buehler). All otoliths were polished until the anti-rostrum became transparent, which indicated that the core was exposed. Following otolith preparation, two classes of geochemical markers were quantified in the otolith cores of yellowfin tuna: trace and minor elements (^7^Li, ^24^Mg, ^55^Mn, ^88^Sr, and ^137^Ba) and stable isotopes (δ^13^C and δ^18^O). Area of otolith cores sampled (i.e., micromill or laser path) for both classes of markers were described previously^15^.

Otolith elemental concentrations were quantified using inductively coupled plasma mass spectrometers (ICP-MS) with laser ablation (LA) systems. Two LA-ICP-MS systems were used over the course of this study: (1) Agilent 7500ce ICP-MS with New Wave 193 nm laser ablation system at the University of Texas at Austin, and (2) XSeries II Thermo Scientific ICP-MS with New Wave 213 nm laser ablation system at Texas A&M University at Galveston. Paired samples were run at both labs to confirm standardization between the two facilities. Five replicate 50 μm diameter circles were ablated in the core region of each otolith, with the first ablation spot placed at the primordium and two spots placed approximately 20 μm apart on each side of the primordium^15^. Otolith material accreted in the area assayed corresponds to approximately the first three months of life based on in-house readings of daily increments. Elemental data from five replicate ablation spots were averaged for each fish.

Time-resolved intensities from the ICP-MS were converted to concentrations (ppm) for trace and minor elements using Iolite software with ^43^Ca as the internal standard Ca index value of 38.3 weight %. Baselines were determined from 30 s gas blank intervals measured while the LA system was off, and all masses were scanned by the ICP-MS. The primary calibration standard used was USGS MACS-3, and the accuracy and precision were assessed using replicates of NIST 612 as an unknown. NIST 612 analyte recoveries were typically within 2% of GeoREM preferred values (http://georem.mpch-mainz.gwdg.de). Element concentrations were then converted to molar ratios (RE, μmol mol^−1^) based on the molar mass of each element (ME, g mol^−1^) and calcium (MCa = 43 g mol^−1^)^23^.

Laser ablation spots were relatively shallow (ca. 30–50 μm) and polished away prior to micromilling core material for stable isotope analysis. A drill path was developed from otolith measurements of small age-0 yellowfin tuna (20–30 cm FL), resulting in core material corresponding to the first 3–4 months of life^15^. Both age-0 and adult yellowfin tuna otoliths were milled to a depth of ca. 700 μm using a 350 mm diameter carbide bit (Brasseler). Stable isotope analysis of powdered core material was performed at the Environmental Isotope Laboratory at the University of Arizona. Otolith δ^13^C and δ^18^O values were quantified using a Finnigan MAT 252 Thermo Fisher Scientific stable isotope ratio mass spectrometer equipped with an automated carbonate preparation device (KIEL-III, Thermo Fisher Scientific). Isotopic ratio measurements were calibrated based on repeated measurements of NBS-18 and NBS-19 (National Bureau of Standards). Otolith δ^13^C and δ^18^O values (‰) are expressed in standard delta (δ) notation as ^13^C/^12^C and ^18^O/^16^O ratios relative to an in-house standard calibrated against Vienna Pee Dee Belemnite.

Multivariate analysis of variance (MANOVA) was used to test whether element:Ca and stable isotope values of age-0 yellowfin tuna differed among the Gulf of Mexico, Caribbean Sea, Cape Verde, and Gulf of Guinea. In addition, univariate contrasts (ANOVAs) were performed on individual element:Ca or stable isotope values to assist in the identification of the most influential markers for discrimination among the four regions. Quadratic discriminant function analysis (QDFA) was utilized to determine the classification accuracy of using otolith geochemistry to assign individual yellowfin tuna to nursery areas. Canonical discriminant analysis (CDA) was used to display multivariate means of geochemical markers for age-0 yellowfin tuna, and discriminant function coefficients were incorporated into CDA plots as vectors from a grand mean to show the discriminatory influence of each marker on discrimination to the four nursery areas in the Atlantic Ocean.

Estimates of nursery origin were determined by comparing geochemical signatures in the otolith cores of adult yellowfin tuna (representing material deposited during age-0 period) to age-0 baseline samples using two assignment methods: HISEA and random forest (RF). HISEA is a conditional maximum likelihood mixed-stock technique^24^ that has been widely used to determine the natal or nursery origin of several species of tunas^8, 25, 26^. Mixture analysis using HISEA included bootstrapping with 1000 simulations to estimate uncertainty around estimated proportions. Individual assignment is increasingly used to assess stock composition, and RF mixture analysis represents a promising machine learning technique for individual assignments of tunas and other taxa^27, 28^. Here, RF was applied without a probability threshold, with assignment to the region with the highest probability. In addition, RF was implemented using a probability threshold of ≥ 0.5 (RF_0.5_), where only individuals with probabilities greater than 0.5 were assigned to a region (nursery). All RF simulations were performed using random Forest package in R^29^. Otolith geochemical baselines used for both HISEA and RF mixture analyses were developed for 2013, 2014, and 2015–2016 cohorts of age-0 yellowfin tuna; no assignment of adult yellowfin tuna was performed using the age-0 baseline from 2017 due to a lack of matched birth years. Individuals from all four nursery areas were not available in 2015 and 2016 and thus the baseline was combined for this period (hereafter 2015–2016 baseline). Also, a limited number of age-0 yellowfin tuna were collected from the Gulf of Mexico during this period, and thus individuals collected in the Gulf of Mexico in 2014 were added to this baseline for HISEA and RF mixture analysis. Assignment of adult yellowfin tuna was based on age-class matching to the correct baseline or birth year.

## Results

Age-0 yellowfin tuna collected from 2013 to 2017 were used as our reference sample (i.e., baseline except for 2017) for investigating the nursery origin of larger, older individuals collected in the WAO. Otolith element:Ca and stable isotope values of age-0 yellowfin tuna varied among the four nurseries, and region-specific signatures were developed using seven geochemical markers: Li:Ca, Mg:Ca, Mn:Ca, Sr:Ca, Ba:Ca, δ^13^C, and δ^18^O (Table 1). Geochemical signatures were significantly different among the four regions for all year classes evaluated (MANOVA, *p* < 0.001). Interestingly, MANOVA results for models developed with only element:Ca ratios or δ^13^C and δ^18^O values were each significant (p < 0.01) across all age-0 baselines tested, signifying the validity of both types of markers for distinguishing yellowfin tuna from the four regional nurseries.

**Table 1:** Mean (+ 1 SD) values for seven geochemical markers (Li:Ca, Mg:Ca, Mn:Ca, Sr:Ca, Ba:Ca, δ^13^C, and δ^18^O) in the otoliths of age-0 yellowfin tuna (*Thunnus albacares*) from the Gulf of Mexico, Caribbean Sea, and two nurseries in the eastern Atlantic Ocean (EAO): Cape Verde and Gulf of Guinea.

Region by year	N	δ^13^C		δ^18^O		Li:Ca		Mg:Ca		Mn:Ca		Ba:Ca		Sr:Ca	
		Mean	SD	Mean	SD	Mean	SD	Mean	SD	Mean	SD	Mean	SD	Mean	SD
2013															
Gulf of Mexico	20	− 9.82	0.40	− 1.72	0.26	5.21	1.00	359.9	55.5	2.94	2.05	1.04	0.46	2194	164
Caribbean Sea	23	− 10.01	0.50	− 1.97	0.18	5.65	0.96	484.3	112.2	3.52	1.76	0.66	0.14	2480	360
EAO-Cape Verde	38	− 9.90	0.40	− 1.70	0.55	4.87	0.80	339.8	58.1	3.21	1.44	0.59	0.19	2312	278
EAO-Gulf of Guinea	21	− 9.70	0.50	− 1.50	0.29	5.15	0.76	384.8	80.8	3.59	1.99	0.64	0.26	2293	228
2014															
Gulf of Mexico	20	− 10.02	0.43	− 1.64	0.20	4.77	0.76	405.6	79.6	2.80	1.39	0.69	0.41	2399	211
Caribbean Sea	26	− 9.59	0.45	− 1.66	0.24	5.29	1.23	401.0	89.3	2.45	1.16	0.57	0.18	2143	189
EAO-Cape Verde	27	− 9.99	0.44	− 1.70	0.24	5.46	1.05	358.4	65.2	3.72	1.46	0.69	0.27	2405	158
EAO-Gulf of Guinea	27	− 9.31	0.37	− 1.72	0.16	6.66	0.64	404.8	59.3	6.27	2.45	0.96	0.34	2292	191
2015-2016															
Gulf of Mexico	22	− 9.99	0.42	− 1.65	0.19	4.87	0.83	405.8	76.6	2.84	1.40	0.71	0.39	2385	210
Caribbean Sea	20	− 9.36	0.59	− 1.69	0.27	6.05	1.09	464.7	97.2	2.76	1.34	0.75	0.22	2052	183
EAO-Cape Verde	30	− 9.69	0.39	− 1.69	0.19	6.48	5.75	379.7	207.9	5.89	2.31	0.92	0.36	2481	309
EAO-Gulf of Guinea	35	− 9.34	0.42	− 1.67	0.21	6.25	1.26	961.9	361.1	5.81	2.38	0.80	0.28	2166	255
2017															
Gulf of Mexico	8	− 10.02	0.52	− 1.87	0.22	7.33	1.62	270.5	46.1	2.92	1.17	1.14	0.71	1953	182
Caribbean Sea	30	− 9.21	0.65	− 1.63	0.25	5.74	0.72	426.2	74.2	4.37	2.15	0.86	0.58	2276	200
EAO-Cape Verde	30	− 9.39	0.47	− 1.75	0.26	7.26	0.80	328.7	51.7	5.01	1.99	0.86	0.43	2170	211
EAO-Gulf of Guinea	30	− 9.31	0.50	− 1.88	0.26	6.92	1.06	375.1	66.9	5.73	2.77	1.83	0.34	2236	149

Samples sizes (N) and mean values are provided for four cohorts (2013, 2014, 2015–2016, 2017) of age-0 fish.

**Figure 2:**
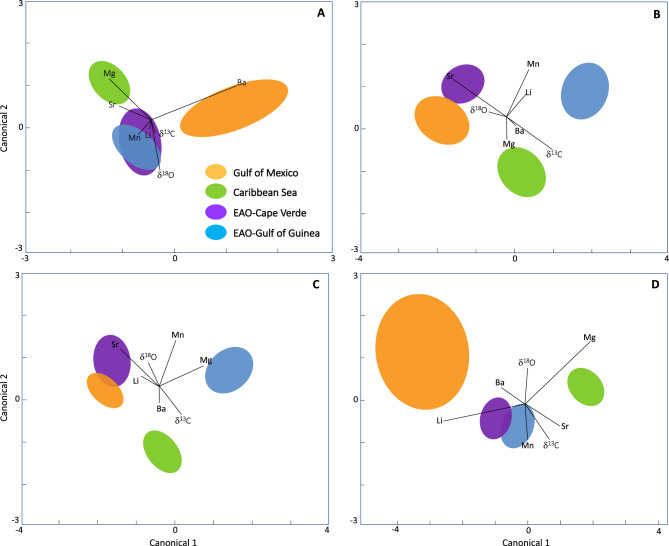
Canonical discriminant analysis (CDA) plots based seven geochemical markers (Li:Ca, Mg:Ca, Mn:Ca, Sr:Ca, Ba:Ca, δ^13^C, and δ^18^O) in the otoliths of age-0 yellowfin tuna (*Thunnus albacares*) from the four primary nurseries in the Atlantic Ocean: Gulf of Mexico, Caribbean Sea, Cape Verde and Gulf of Guinea; eastern Atlantic Ocean (EAO) denoted for the two nurseries in this region. Plots shown for four cohorts of age-0 yellowfin tuna: (**A**) 2013, (**B**) 2014, (**C**) 2015-16, (**D**) 2017. Samples sizes of age-0 fish used for each region and year shown in Table 1. Ellipses represent 95% confidence limit around each multivariate mean and biplot vectors show the influence of each geochemical marker on regional discrimination.

Univariate contrasts indicated that several of the geochemical markers investigated were influential in distinguishing age-0 yellowfin tuna from the four nursery areas (Fig. 2). Region-specific mean element:Ca and stable isotope values often varied from year to year for age-0 yellowfin tuna and consistent regional differences were generally not observed across all cohorts assessed (Table 1). Nevertheless, some notable trends in otolith geochemistry persisted across multiple year classes of age-0 yellowfin tuna for Mn:Ca, Sr:Ca, Ba:Ca, and δ^13^C values (Fig. 2). In particular, mean otolith Mn:Ca for age-0 fish from the Gulf of Mexico and Caribbean Sea across all collection years were often significantly lower (3.6 and 3.3, respectively) than values observed in the two regions in the EAO (Gulf of Guinea and Cape Verde > 4.5). Similarly, mean otolith Sr:Ca ratios were lower for age-0 yellowfin tuna from the Gulf of Mexico (2182) relative to the three other regions across all years, which ranged from 2237 to 2346 (Table 1). Conversely, otolith Ba:Ca ratios were high in both the Gulf of Mexico (0.96) and Gulf of Guinea (0.90) relative to the two other regions (< 0.80). For stable isotopes, otolith δ^13^C values were often lowest in the Gulf of Mexico relative to all other collection regions.

Overall cross-validated classification success of age-0 yellowfin tuna from QDFA varied across the year classes assessed but overall classification to each region was relatively high among the year classes investigated: 2013 (67.3%), 2014 (89.0%), 2015–2016 (93.4%), and 2017 (81.6%). Overlap in canonical scores (ellipses) and misclassifications were often associated with samples from the two regions in the EAO (Fig. 2). In fact, age-0 baselines with the lowest observed overall classification success (i.e., 2013, 2017) were primarily due to misclassification between age-0 yellowfin tuna collected in the Gulf of Guinea and Cape Verde. Our ability to distinguish age-0 yellowfin tuna collected from these two areas for the 2013 and 2017 baselines relative to the Gulf of Mexico and Caribbean Sea increased to 89.4% and 90.8%, respectively, when both regions in the EAO were pooled.

**Figure 3:**
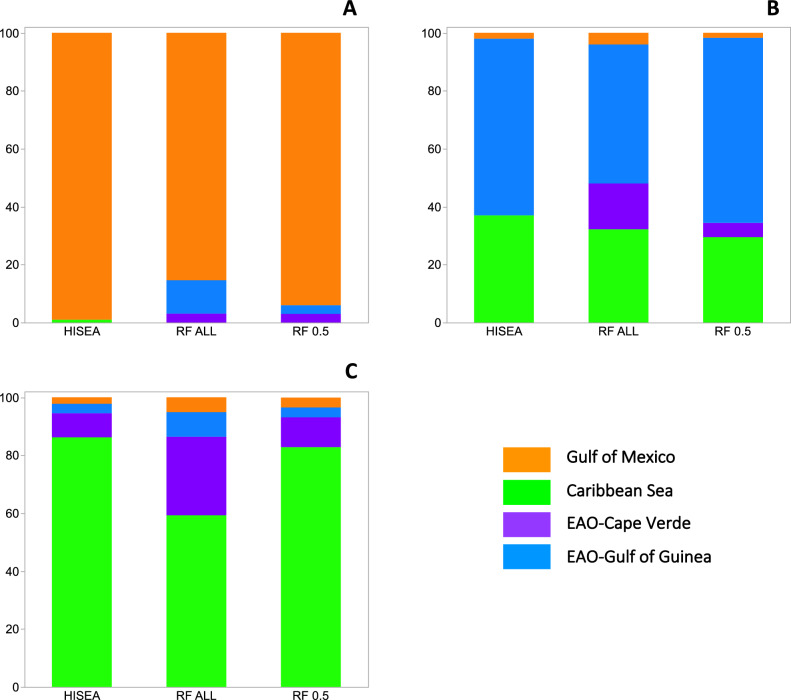
Mixed-stock predictions of the nursery origin for sub-adult and adult yellowfin tuna (*Thunnus albacares*) in the Gulf of Mexico. Percent contribution estimates from HISEA and random forest (RF) assignment methods. RF ALL represents random forest classification to the region with the highest probability (no samples excluded) while results from RF 0.5 is limited to individual assignment where the probability of assignment to one region is ≥ 50%. Percent contribution estimates to each of the four nurseries (Gulf of Mexico, Caribbean Sea, Cape Verde, Gulf of Guinea) is shown for sub-adult and adult yellowfin tuna based on their birth year(s): (**A**) 2013 (n = 96), (**B**) 2014 (n = 152), (**C**) 2015 (n = 59). Individuals from the two nurseries in the eastern Atlantic Ocean (EAO) represent trans-Atlantic migrants to this fishery.

**Figure 4:**
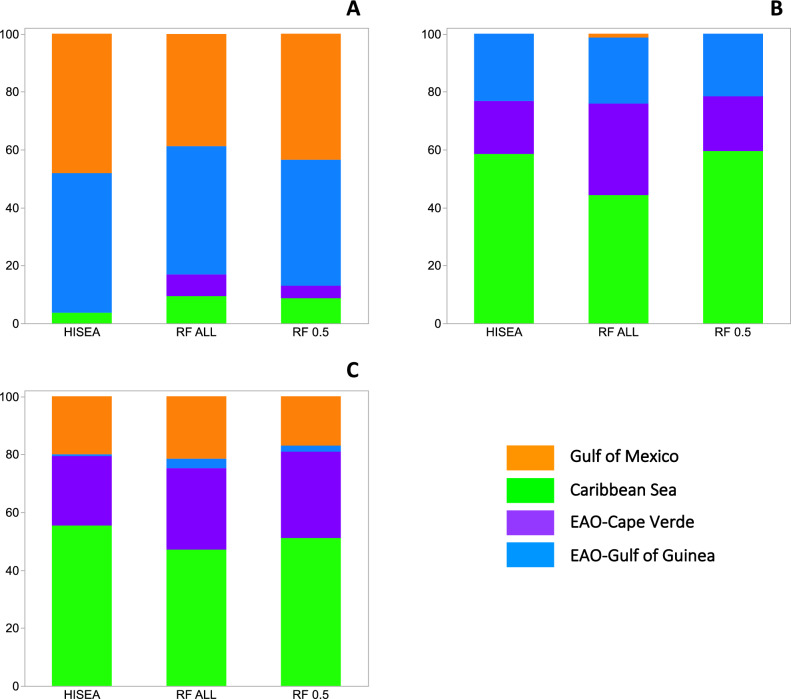
Mixed-stock predictions of the nursery origin for sub-adult and adult yellowfin tuna (*Thunnus albacares*) in the Mid Atlantic Bight. Percent contribution estimates from HISEA and random forest (RF) assignment methods. RF ALL represents random forest classification to the region with the highest probability (no samples excluded) while results from RF 0.5 is limited to individual assignment where the probability of assignment to one region is ≥ 50%. Percent contribution estimates to each of the four nurseries (Gulf of Mexico, Caribbean Sea, Cape Verde, Gulf of Guinea) is shown for sub-adult and adult yellowfin tuna based on their birth year(s): (**A**) 2013 (n = 106), (**B**) 2014 (n = 79), (**C**) 2015 (n = 121). Individuals from the two nurseries in the eastern Atlantic Ocean (EAO) represent trans-Atlantic migrants to this fishery.

Nursery origin of adult yellowfin tuna based on age-class matching to their baseline was performed on over 600 individuals collected from the Gulf of Mexico and Mid Atlantic Bight over a three-year period. Maximum likelihood estimates of nursery origin derived with HISEA indicated that local production in the Gulf of Mexico was highly variable across cohorts assessed. The Caribbean Sea and both areas in the EAO were responsible for meaningful contributions of yellowfin tuna recruits to WAO fisheries in the Gulf of Mexico (Fig. 3) and Mid Atlantic Bight (Fig. 4). Adult yellowfin tuna in the 2013 cohort (birth year) caught in the Gulf of Mexico and Mid Atlantic Bight were assigned largely to the Gulf of Mexico (98.9 ± 1.0% and 48.1 ± 6.4%, respectively; mean ± 1SD), with trans-Atlantic migrants from the Gulf of Guinea also making up a significant fraction of the yellowfin tuna in the sample from the Mid Atlantic Bight (48.2 ± 7.1%). Similarly, trans-Atlantic migrants of yellowfin tuna from the Gulf of Guinea were also detected in large numbers in the 2014 cohort collected in the Gulf of Mexico (61.6 ± 10.1%) and Mid Atlantic Bight (23.2 ± 8.6%). In addition, migrants from the Caribbean Sea were well represented for yellowfin tuna with 2014 and 2015 cohorts caught in the Gulf of Mexico (36.6 ± 10.0% and 86.2.2 ± 5.9%, respectively) and the Mid Atlantic Bight (58.6 ± 9.1% and 55.4 ± 6.2%, respectively), accounting for about half of the yellowfin tuna in both WAO fisheries.

Individual assignment using RF without any probability threshold (i.e., all individuals assigned to region based on the highest probability) produced relatively similar contribution estimates to HISEA-based predictions (Figs. 3, 4). Again, a significant fraction of the adult yellowfin tuna in the 2013 cohort (birth year) caught in the Gulf of Mexico and Mid Atlantic Bight were assigned to the Gulf of Mexico (85.4% and 38.7%, respectively), with a modest percentage of trans-Atlantic migrants from the Gulf of Guinea present in the Gulf of Mexico (11.5%) and Mid Atlantic Bight (44.3%) samples. Trans-Atlantic migrants from the Gulf of Guinea were also detected in large numbers in the 2014 cohort collected in the Gulf of Mexico (71.6%), with the Mid Atlantic Bight displaying a high percentage of migrants from the EAO (54.4%): Gulf of Guinea (22.8%), Cape Verde (31.6%). In addition, migrants from the Caribbean Sea were well represented for yellowfin tuna from the 2014 cohort caught in the Gulf of Mexico (32.2%) and Mid Atlantic Bight (44.3%). For adult yellowfin tuna in the 2015 cohort, the percentage of WAO recruits (i.e., Gulf of Mexico) detected in Gulf of Mexico and Mid Atlantic Bight samples were limited (5.0% and 21.5%, respectively), with the largest fraction again predicted to originate from the Caribbean Sea. Pooling individual assignments across the three cohorts and combining assignments from both EAO regions (Gulf of Guinea, Cape Verde) resulted in similar estimates of nursery origin of adult yellowfin tuna in both U.S. fisheries in the WAO: Gulf of Mexico (Gulf of Mexico = 29.6%, Caribbean Sea = 27.4%, EAO = 43.4%) and Mid Atlantic Bight (Gulf of Mexico = 22.1%, Caribbean Sea = 33.2%, EAO = 44.3%).

Random forest was also used to estimate proportions (percentages) to each nursery using only individuals with a threshold probability of > 0.5 (RF_0.5_) to a specific nursery. The addition of the 0.5 threshold probability reduced the overall number of individuals that could be assigned to a region by approximately 50% (310 of 613). Although based on smaller sample sizes, individual assignments of yellowfin tuna with RF_0.5_ were comparable to mixing rates from HISEA and RF without a probability threshold (Figs. 3, 4). Using RF_0.5_, adult yellowfin tuna in the 2013 cohort (birth year) from the Gulf of Mexico were again predicted to originate primarily from this region (94.0%) with migrants from the Gulf of Mexico also contributing to the fishery in the Mid Atlantic Bight (43.4%). A notable percentage of trans-Atlantic migrants from the Gulf of Guinea were present in the Mid Atlantic Bight (43.3%) for the 2013 cohort. Trans-Atlantic migrants from the Gulf of Guinea were again well represented in the Gulf of Mexico sample for the 2014 cohort (63.9%). Migrants from the Caribbean Sea were again common in the 2014 cohort of adult yellowfin tuna samples from the Gulf of Mexico (29.5%) and Mid Atlantic Bight (59.4%). In the Mid Atlantic Bight, most of the remaining individuals were predicted to be of EAO (Gulf of Guinea or Cape Verde) origin (40.5%). For adult yellowfin tuna in the 2015 cohort, RF_0.5_ predicted that a considerable percentage of yellowfin tuna in both the Gulf of Mexico and Mid Atlantic Bight were of Caribbean Sea origin (82.7% and 51.0%, respectively). Pooling individual assignments across the three cohorts using RF_0.5_ again showed that EAO and Caribbean Sea nurseries were important contributors of yellowfin tuna to fisheries in the WAO: Gulf of Mexico (Gulf of Mexico = 41.4%, Caribbean Sea = 26.8%, EAO = 31.8%) and Mid Atlantic Bight (Gulf of Mexico = 24.8%, Caribbean Sea = 34.0%, EAO = 41.2%).

## Discussion

Region-specific variability was present for several of the geochemical markers quantified in the otoliths of age-0 yellowfin tuna, and spatial trends were coupled with temporal (interannual) differences within regional nurseries. Pronounced spatial and temporal changes in otolith geochemistry at ocean-basin scales have been reported for both temperate and tropical tunas in the Atlantic Ocean^25, 30, 31^, Pacific Ocean^8, 9, 32^ and Indian Ocean^33, 34^. Year-to-year changes in the relative importance of geochemical markers for region-specific discrimination at this scale are often driven by seasonal or annual variation in coastal and oceanographic processes that alter ambient seawater conditions^15, 23, 35^. Further, the use of region-specific geochemical markers is complicated by the fact that yellowfin tuna and other highly migratory species range widely, often within annual migration cycles, and regularly cross oceanographic provinces and mesoscale oceanographic features that have different physicochemical properties^23, 36, 37^. Despite the potential limitations of the natural tags used in the present study, regional patterns were detected for several influential markers (e.g., Mn:Ca, Sr:Ca, Ba:Ca, δ^13^C), allowing for the discrimination of age-0 yellowfin tuna from the four nurseries.

Temporal variability in the geochemistry of otoliths from age-0 yellowfin tuna prevented us from applying an integrated baseline (pooled across multiple cohorts or years within a region) to determine the origin of adult yellowfin tuna in WAO fisheries. This was due in part to the use of two different types of geochemical markers: trace elements and stable isotopes. Previous research has demonstrated a fair degree of temporal stability for stable isotope values (e.g., δ^18^O) in the otoliths of yellowfin tuna and bigeye tuna (*T. obesus*) in the Pacific Ocean^9^ and bluefin tuna (*T. thynnus*) in the Atlantic Ocean^10^, while many element:Ca ratios are often variable from year-to-year within the same region^8^. Here, both classes of geochemical markers were required to effectively discriminate age-0 yellowfin tuna from the four regional nurseries and the inclusion of more temporally variable otolith element:Ca ratios required age-class matching for individuals of unknown origin to their respective baseline (i.e., matching birth year). While age-class matching is the preferred approach when baseline signatures vary across time within the same region, potential errors in assignment of age (birth year) to the proper baseline sample can lead to uncertainty in assignment to nursery areas, particularly when intrinsic (i.e., within individual) variability of geochemical markers is high.

**Figure 5:**
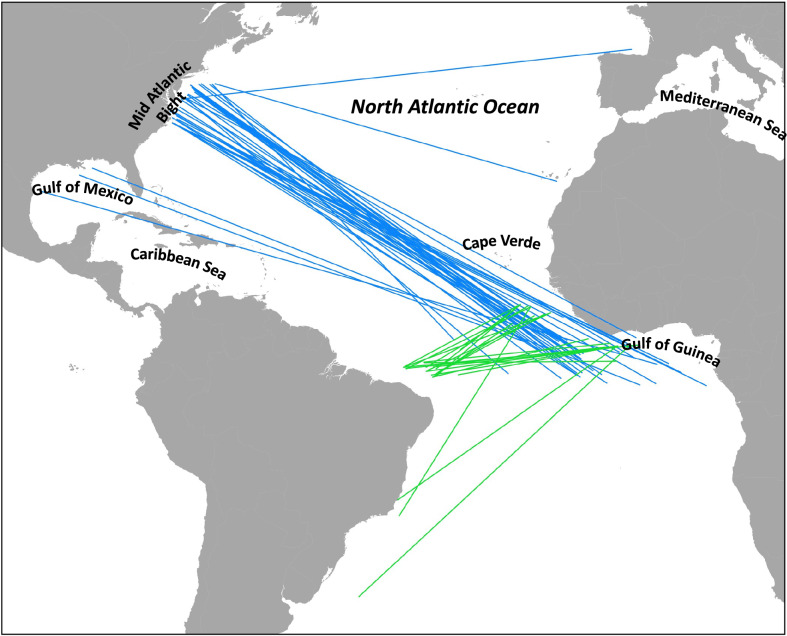
Summary of tag releases and recaptures of yellowfin tuna (~ age-0 to age-3 +) from ICCAT’s conventional tagging database that display trans-Atlantic migrations (defined here as longitudinal shifts greater than 1000 km). Blue and green lines represent release locations in WAO (west to east migration) and EAO (east to west migration), respectively. Access to ICCAT statistical database: https://www.iccat.int/en/accesingdb.html. Map generated with QGIS (V. 3.32, www.qgis.org).

Estimates of nursery origin for adult yellowfin tuna in Gulf of Mexico and Mid Atlantic Bight fisheries varied markedly across birth years investigated, underscoring the dynamic nature of trans-Atlantic movements and stock mixing. Although the stock (nursery) composition of adult yellowfin tuna varied from year-to-year, results clearly demonstrate that the contribution of recruits from outside production zones in the Caribbean Sea and off the coast of Africa support both fisheries in the WAO. The identification of trans-Atlantic migrants in U.S. waters from nurseries in the Gulf of Guinea and the Cape Verde archipelago is not surprising given that recaptures of tagged sub-adult and adult yellowfin tuna indicate that this species commonly migrate thousands of kilometers from release locations, often crossing the Atlantic Ocean^3, 13^. Moreover, recaptures from ICCAT’s conventional tag database clearly demonstrates trans-Atlantic migrations by yellowfin tuna with recruits of eastern and western origin commonly mixing (Fig. 5). Although yellowfin tuna tagged in the Gulf of Guinea or Cape Verde were not commonly recaptured in the WAO, large numbers of fish tagged in the Mid Atlantic Bight were recaptured in the Gulf of Guinea. This pattern is symbolic of natal homing back to their place of origin, and trans-ocean return migrations to natal areas have been reported for other true tunas^38, 39^.

In addition to large-scale trans-Atlantic (east to west) migrations, the presence of large numbers of yellowfin tuna of Gulf of Mexico or Caribbean Sea origin in the WAO indicates the existence of several different migratory contingents within the Atlantic-wide population. In fact, the presence of locally produced yellowfin tuna in the Gulf of Mexico indicated that a fraction of the population displays resident behaviors or more localized movements, which has been confirmed for yellowfin tuna in the Gulf of Mexico with satellite tags^4, 10, 40^. The coexistence of two or more migratory contingents with contrasting movement behaviors (e.g., resident to trans-Atlantic) is indicative of a partial migration type of life history strategy, which may lead to greater resilience and stability of the Atlantic-wide population^41^. It is also important to note that fish from both the Gulf of Mexico and Caribbean Sea also support the Mid Atlantic Bight fishery, further demonstrating that partial migration behaviors may vary among individuals produced in the different nurseries or production zones, with scales of movement (maximum displacement distances) and migration pathways being influenced by an individual’s place of origin. Population connectivity of yellowfin tuna between U.S. waters in the WAO and the Caribbean Sea has also been demonstrated through conventional tagging^3^ but the degree of connectivity with the Caribbean Sea was assumed to be relatively limited. Our findings suggest that recruits from the Caribbean Sea are important to both Gulf of Mexico and Mid Atlantic Bight fisheries.

Assignment methods applied to determine the nursery origin of adult yellowfin tuna—both mixture (HISEA) and individual random forest (RF, RF_0.05_) classifications—resulted in modest differences in stock structure by year for fish captured in both WAO fisheries. However, overall trends in nursery origin of adult yellowfin tuna in both the Gulf of Mexico and Mid Atlantic Bight were generally similar among the three assignment approaches. Mixture analysis such as HISEA is often preferred over individual assignment methods because the latter approach combines individual assignments to generate estimated proportions of stocks, which can lead to greater uncertainty and bias^28, 42^. Nevertheless, individual assignment methods are increasingly used to assign individuals to their stock or place of origin^6, 43, 44^. Individual assignment such as RF allows scientists to retrospectively determine an individual’s nursery origin, which can then be used to partition samples by age, size, sex, or time of collection to further investigate factors influencing migratory behaviors^27^. Our finding of comparable contribution rates by nursery among the three assignment methods is in accordance with other studies that have reported generally consistent estimates between mixture analysis and individual assignment methods^27, 45^. While general trends were consistent and not overly sensitive to the different assignment methods applied, it is important to mention that predictions were highly sensitive to the baseline used for both HISEA and RF.

High levels of mixing by yellowfin tuna from the different production zones and strong interannual variation in region-specific contribution rates highlight the complex and dynamic nature of this species’ stock structure. In addition, geographic shifts in the distribution of yellowfin tuna across national boundaries lead to governance and management challenges, which are further exacerbated by the fact that mixing rates of this species in the WAO appear to be highly variable. The prevalence of significant numbers of migrants from Caribbean Sea and EAO production zones suggests that U.S. fisheries in the WAO are heavily subsidized in some years from outside production zones. Given the reliance of WAO fisheries on production zones in the EAO, management measures that mitigate bycatch mortality of small yellowfin tuna in purse-seine fisheries in the EAO will benefit fisheries throughout the Atlantic Ocean, including U.S. fisheries. Furthermore, high levels of east to west exchange and mixing support ICCAT’s single stock assumption for yellowfin tuna in the Atlantic Ocean^13^; however, it is important to note that the origin of harvested fish (i.e., nursery-specific contribution) will have important management implications, with shifting distributions leading to more uncertainty and less reliable population projections^46^. As a result, there is a clear need to further resolve migration rates and pathways, residence times, population connectivity, and changing productivity of yellowfin tuna from the different spawning and/or nursery areas. Such knowledge is essential for developing conservation strategies that adapt to climate change and improve the resilience of yellowfin tuna populations and associated ecosystems in the Atlantic Ocean.

### Supplementary Information

Supplementary Table S1.

## Data Availability

Geochemical data for age-0 and adult yellowfin tuna by region and year are available in Supplementary Data File S1. Additional details regarding dataset used in the current study are available from the corresponding author upon request.
